# New dry technology of environmentally friendly biomass refinery: glucose yield and energy efficiency

**DOI:** 10.1186/s13068-014-0138-2

**Published:** 2014-09-24

**Authors:** Abdellatif Barakat, Xavier Rouau

**Affiliations:** INRA, UMR 1208 Ingénierie des Agropolymères et Technologies Emergentes 2, Place Pierre Viala, 34060 Montpellier, cedex 1 France

**Keywords:** Wheat straw, Biorefinery, Milling and electrostatic fractionation, Biofuels

## Abstract

**Background:**

Today, most of pretreatments used to convert biomass into biofuels are based on expensive chemical processes that not only do not keep the major components intact after separation, but also consume water and generate many effluents. However, dry fractionation technologies are an important step for future biomass biorefineries since they do not require chemicals and do not generate wastewater. Therefore, the aim of the present study was to evaluate the feasibility of using milling combined with an electrostatic fractionation (ES) of wheat straw (WS) as a way to separate fractions that are enriched in cellulose and more enzymatically accessible, from recalcitrant tissues enriched in lignin-hemicelluloses, in order to produce biofuels.

**Results:**

After milling, WS particles are introduced into a tribo-electrostatic separator, where they are positively or negatively charged by tribo-electricity. Then they are introduced into a separation cell comprising two electrodes (+ and –). The negative electrode attracts the positively charged particles and the positive electrode attracts the negatively charged particles. Results show that amorphous cellulose rich particles were clearly more abundant in positively charged fractions (F+), and loose crystalline cellulose, lignin-xylan and ash-containing material were more abundant in negatively charged fractions (F–). Indeed, positively charged fractions (F+) are more accessible upon enzymatic hydrolysis, which resulted, for example, in sugars yield of 43.5% glucose (254 gKg^−1^) for F2B + compared to 25.2% (103 gKg^-1)^ for F2A–, and 26.3% (130 gKg^−1^) for unfractionated WS F0, respectively.

**Conclusions:**

The combination strategy of milling and ES fractionation could improve the economic feasibility by low energy consumption (10.5 WhKg^−1^) and it produces reactive lignocelluloses particles with different physicochemical structures, which can be converted easily into biofuels and biomaterials without generating toxic effluents.

## Background

Lignocellulosic plant cell walls consist mainly of cellulose, hemicelluloses and lignin. These polymers together with small amount of other components, like acetyl groups, minerals, proteins and phenolic substituents, are organized in complex three-dimensional structures, which are not uniform for different plants. Moreover, lignocellulosic plants consist of different botanical parts of various tissues present in different proportions, each tissue having its own physical properties and biochemical compositions [1–3]. In a biomass biorefinery situation, the separation of lignocellulose into its major tissues and components constitutes the first step of its refining to high-value-added products [4,5]. Today, most of lignocellulosic fractionation technologies or pretreatments are based on expensive chemical processes (pulping, acidic hydrolysis, solvent extraction, alkaline and acid extraction, steam and ammonia explosion and so on) which do not keep the major components intact after separation and generate high toxic effluents [6]. Achieving high fractionation yields and maintaining the integrity of the macromolecular fractionation products without using a high quantity of chemicals and without generating wastewater are of major importance, regarding the effectiveness of the refining process.

Mechanical or dry fractionation could deconstruct the lignocellulosic biomass at different levels of the plant [7–9]. The development of a dry separating operation combined milling step can therefore allow the separation of parts of plants, tissues and cells and the acquisition of different enriched fractions with different physical and physicochemical properties. A dry fractionation or refinery of lignocellulosic biomass without using chemical and water could allow to produce different tissues enriched in cellulose, hemicelluloses and/or lignin and increase the efficiency of processes while reducing the associated costs and effluent production [7,10]. These processes are based on a combination of fragmentation and separation steps, which are carried out by a mechanical pretreatment followed by several types of fractionation technologies [11–13].

Papatheofanous *et al*. [12] developed a dry fractionation of yellow winter wheat straw (WS), which was initially milled by a disc mill and separated into two fractions by sieving on a 1 mm screen - chips containing mostly internodes and meal consisting mainly of ground leaves and nodes [12]. The authors showed that all internodes passed in the chip fraction and that the internode fraction (63% of the whole straw) contained 8% more cellulose, 9% more lignin and 10% less ash than the unfractionated material [12]. Chundawat *et al*. [14] also showed that corn stover fractions with a high corn leaf content were found to be more susceptible to enzymatic hydrolysis [14]. Zhu *et al*. [15] reported that the very fine sample (<0.127 mm) has a completely different chemical composition as compared to those of the rest of samples. The fine fraction contains about 40% more lignin and 33% less cellulose than the rest of the fractions. Furthermore, this sample has a much larger surface area and is more susceptible to enzymatic hydrolysis. Fractionation of fibrous fraction from steam-exploded rice straw (SERS) with a high moisture content has been studied with respect to the separation degree of fibrous tissue versus non-fibrous tissue including epidermal, parenchyma and vessel tissue using a fluidized bed opposed jet mill [9]. Chemical composition and fiber characteristics, such as fiber size and composed cell proportion, were studied for the separated fibrous fraction of SERS. A high amount of cellulose fibrous fraction (70.4%), with 63.1% fiber cell content and 65.6% cellulose content, was produced from the fractionation process. The fiber characteristics of this fractionated fibrous fraction were: mean length (0.97 mm), mean width (8.6 μm), fiber cell (63.1%), parenchyma cell (33.5%), epidermis cell (2.4%) and vessel cell (1%). The fluidized bed opposed jet mill method is suitable for producing high-fiber tissue content fractions without extensively damaging the raw fibers [9].

**Figure 1:**
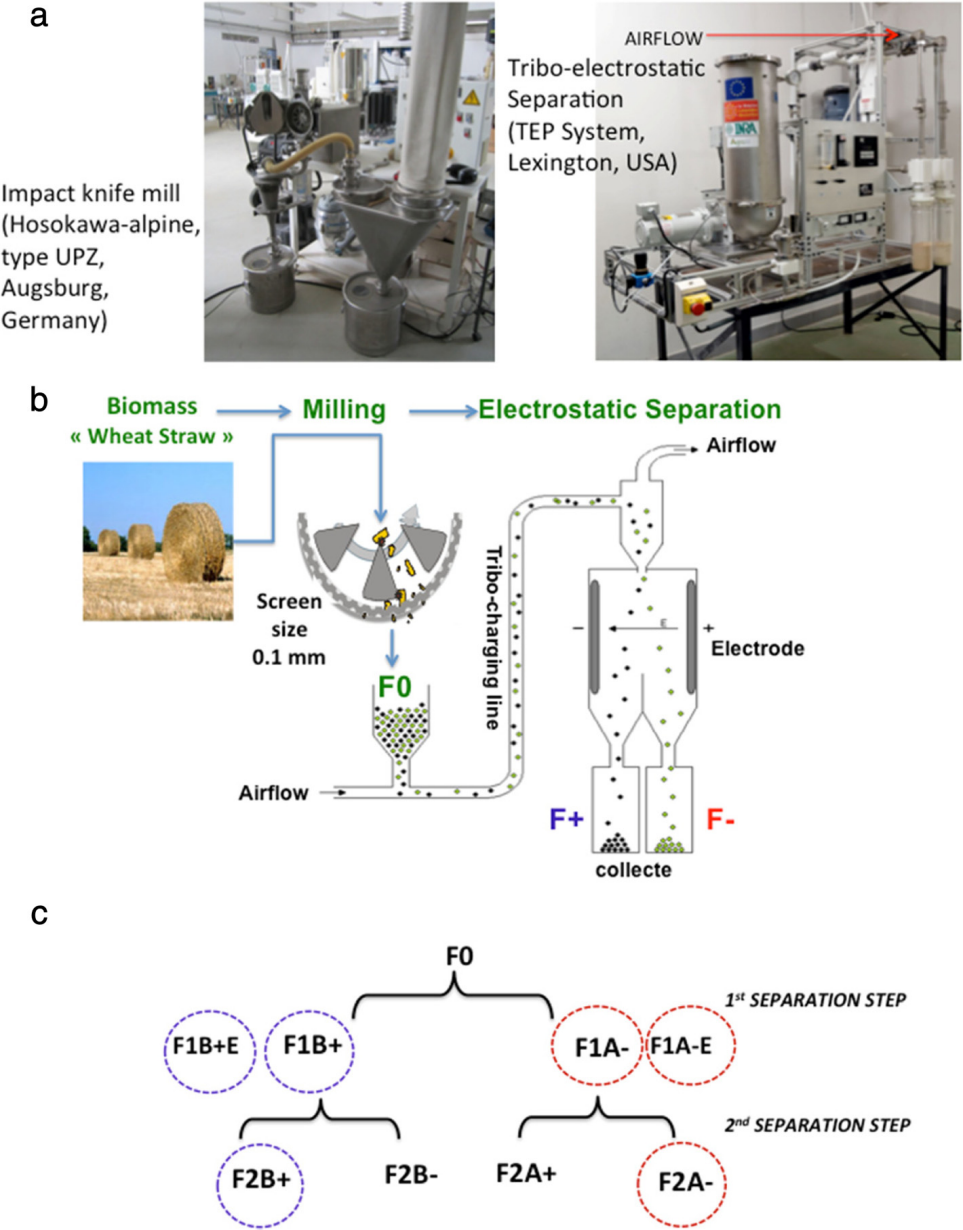
**Innovative dry fractionation biorefinery scheme developed in this study using the combination of milling and electrostatic separation: a)** Photo of impact mill pilot (type UPZ, Hosokawa-alpine, Augsburg, Germany) and Tribo-electrostatic Separator Pilot (TEP Systems, Lexington, United States) used in this study; **b)** Dry fractionation route and principal of electrostatic separation; **c)** Fractionation scheme and preparation of electrostatic fractions.

The combination of low-severity steam explosion and superfine grinding has been studied with respect to side products generation and enzymatic hydrolysis efficiency [9,16]. The superfine ground product hydrolyzed at the highest rate produced the most elevated levels of reducing sugars (61.4%) after 24 hours of hydrolysis. This is 2.8 and 2.3 times higher than those from coarsely pulverized and steam-exploded rice straw, respectively. Hemery *et al*. [11,17] fractionated wheat bran by combination of ultrafine dry milling and electrostatic separation. The objective was to break down bran tissues in order to separately isolate their sub-cellular constituents (cell walls rich in fiber versus cell content rich in micronutrients). In this case, particles are conveyed by compressed air in a charging line where they are charged by tribo-electricity (Figure 1a and b). This type of separation was notably used successfully to prepare fractions concentrated in aleurone and pericarp from wheat bran [11,17]. The authors showed that fiber-rich particles of pericarp were more abundant in the fractions of negatively charged particles, and aleurone cell walls (β-glucans, arabinoxylans and ferulic acid) and loose protein containing material from aleurone and endosperm was more abundant in the positively charged particles.

**Figure 2:**
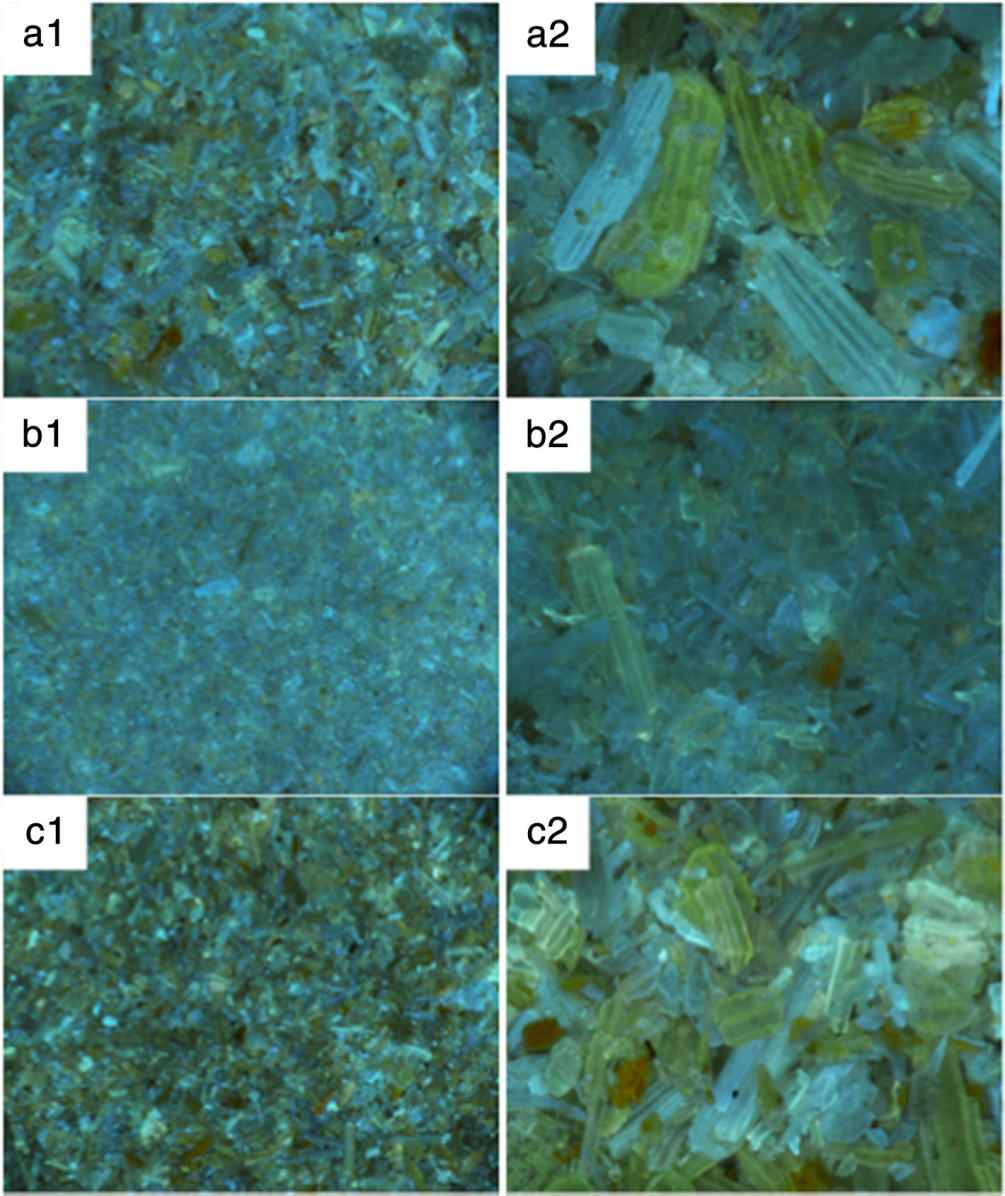
**Micrographic and morphology of unfractionated WS**: **a1)** × 5 and **a2)** × 25, Positively charged fractions: **b1)** × 5 and **b2)** × 25, and negatively charged fractions **c1)** × 5 and **c2)** × 25. WS: Wheat straw.

The aim of the present study was to evaluate the potential of using ultrafine grinding followed by electrostatic fractionation as a way to obtain or separate different tissues enriched in cellulose and/or lignin-hemicelluloses from WS for biofuels and biomaterials production. This biorefining approach suggested the combination of two dry mechanical technologies (Figure 1): 1) Ultrafine milling with the aim of fragmenting and dissociating different lignocellulosic tissues and components (Figure 1a and b); 2) Electrostatic separation (ES) based on the preloading of particles with a subsequent separation in an electrical field (Figure 1a and b).

The influence of the fractionation steps on the biochemical composition, particle size and microstructure were studied, and the interest of the different fractions was evaluated.

## Results and discussion

### Structural properties and biochemical composition of wheat straw fractions prepared by electrostatic separation technology

On an industrial scale, electrostatic separation (ES) is used as a technology for electric or polymers waste separation. For instance, ES can separate efficiently different polymers with various physicochemical properties (polyvinyl chloride, polyethylene and so on) or metals from polymers [18,19]. In our laboratory we developed this technology with the objective to perform a dry fractionation lignocellulose biorefinery without effluent generation. Dry fractionation of WS, developed in this study, is a combination of two different technologies (Figure 1a and b). One is a structural deconstruction and dissociation of the plant cell wall tissues and macromolecules by ultrafine milling (knife milling with a screen size of 0.1 mm). The other one is an electrostatic separation (ES) or fractionation in order to isolate and separate different tissues or fractions, according to their biochemical composition and surface properties. For this purpose, two successive steps of electrostatic fractionation were carried out, using WS powder (with 0.1 mm screen size), as the starting material F0 (Figure 1b and c). Samples were collected after each separation step, yielding eight fractions (Figure 1c). The particles adhering to the electrodes were also collected but only at the first step named F1A–E and F1B+E fractions. F0, F1A–, F2A–, F2B–, F1A–E, F1B+, F2B+, F2A+ and F1B+E samples were analyzed for their particle size; color, biochemical composition, crystallinity and microstructure and enzymatic accessibility. The yield of different fractions is given in Table 1. The general characteristics and physicochemical properties of lignocellulosic materials studied are also summarized in Table 1.

**Table 1:** **Biochemical composition and physicochemical properties of different WS fractions**

**Fractions**	**Recovery (%)** ^**a**^	**D**_**50**_ **(μm)** ^**b**^	**(% w/w)** ^**b**^				**Hem:Cell**	**CrI (%)** ^**a**^	**SA (m** ^**2**^ **/g)**
			**Ash**	**Lignin**	**Hem**	**Cell**			
**F0**	-	81.9 ± 3.4	4.5 ± 1.1	21.5 ± 1.3	29.1 + 1.2	45.4 ± 2.4	0.66	54.9 ± 0.8	43.6
**F1A–**	35 ± 4.2	81.2 ± 2.2	5.1 ± 0.8	22.4 ± 1.7	31.6 ± 1.7	40.9 ± 2.0	0.77	58.8 ± 0.0	42.5
**F1A–E**	4 ± 1.2	42.2 ± 1.2	15.3 ± 2.2	16 .7 ± 2.2	30.3 ± 0.8	37.6 ± 0.9	0.81	63.5 ± 2.2	70.7
**F2A–**	22 ± 2.6	95.7 ± 2.7	5.2 ± 1.3	21.3 ± 0.9	32.6 ± 1.5	40.8 ± 1.4	0.82	60.4 ± 1.7	37.3
**F2B–**	28 ± 4.5	75.8 ± 1.8	4.8 ± 0.9	20.9 ± 2.2	29.1 ± 0.8	45.2 ± 1.1	0.63	60.1 ± 0.0	47.1
**F1B+**	58 ± 4.6	52.2 ± 3.1	3.7 ± 0.8	18.3 ± 1.1	22.8 ± 1.7	55.2 ± 1.6	0.45	51.9 ± 1.1	60.2
**F1B+E**	5 ± 1.3	44.9 ± 0.7	2.9 ± 0.4	16.3 ± 1.3	21.8 ± 1.1	58.9 ± 1.2	0.37	51.3 ± 2.1	76.4
**F2B+**	32 ± 5.2	62.9 ± 1.2	2.6 ± 0.4	17.7 ± 0.8	21.7 ± 0.8	58.4 ± 0.8	0.37	52.3 ± 0.7	63.2
**F2A+**	16 ± 2.6	55.5 ± 1.5	3.5 ± 1.0	19.5 ± 1.2	24.6 ± 1.2	52.5 ± 1.3	0.49	55.8 ± 1.2	59.4

^a^in duplicate; ^b^in triplicate.

WS: wheat straw; D_50_: median size; SA: Surface Area; Cell: Cellulose; Hem: Hemicelluloses; CrI: Crystallinity index.

SA = (Sp*/*Vp*)/*ρ.

Sp = surface of particle (m^2^) = 4π(*(*Zp*/2)* ^*2*^ *); Z*p: Particle size (m).

Vp = volume of particle (m^3^) = 4/3π((Zp/2)3).

ρ: density of particle (g/m^3^).

Results show that the biochemical and physicochemical properties of the different fractions produced varied according to the charge of particles in the fractions and the number of separation steps carried out. The fluorescence microscopy analyses showed that the positively charged fractions F+ were bluer than the negatively charged fractions F– (more brownish), whereas the starting material F0 is a mixture of two fractions. Figure 2 shows that the morphology of the positively charged fractions differed from negatively charged fractions and unfractionated WS (F0). The positively charged fractions contain homogeneous and non-fibrous small particles. Conversely, the negatively charged fractions contain more heterogeneous and fibrous long particles. Hemery *et al*. [11] obtained similar results with wheat bran after successive ES. This difference in color and morphology could be due to the difference in composition, depending on the origin of the tissues. The separation process also influenced the particle median diameter D_50_. Indeed it was observed that at each separation step, the positively charged F+ fractions were composed of finer particles than the corresponding negatively charged F– fractions (Table 1). Except the fractions adhering to the electrodes, F1A–E (42.2 μm) fraction is finer compared to F1B+E fraction with D_50_ of 44.9 μm. ES leads to a particle size of 52.2 μm and 62.6 μm for F1B+ and F2B+, respectively compared to 81.2 μm, 95.7 μm and 81.9 μm for F1A–, F2A– and F0, respectively (Table 1). The surface area (SA) varied also according to charge of particles (Table 1). Generally, the positively charged fractions exhibited a larger surface compared to negatively charged particles except F1A–E. It can be seen in Table 1 that ES leads to a SA of 42.5, 37.3 and 47.1 m^2^/g for F1A–, F2A– and F2B–, respectively compared to 43.6 m^2^/g for F0 and 60.2, 63.2 and 59.4 m^2^/g for F1B+, F2B+ and F2A+, respectively.

Miao *et al*. [20] investigated the mechanical size reduction of miscanthus and switchgrass using a commercial hammer mill and the resulting SA was measured using the geometry and density of particles. This method yielded accessible SA of 20.4 and 20.5 m^2^/g when a hammer mill was used to grind miscanthus and switchgrass, respectively, with a screen size of 1 mm [20]. They reported that, in general, the SA increases linearly with particle size. Literature data reported that a decrease in particle size and an increase of reactive SA could facilitate the process of enzymatic hydrolysis [10,21]. ES also influenced cellulose crystallinity. It was observed that the negatively charged fractions F– exhibited higher CrI compared to F0 and positive fractions F+. It can be seen in Table 1 that ES leads to a CrI of 58.8, 63.5 and 60.4% for F1A–, F1A–E and F2A–, respectively compared to 51.9, 51.3 and 52.3% for F1B+, F1B+E and F2B+. It is widely accepted that crystalline cellulose is less accessible to cellulase attack than amorphous cellulose [22–25].

**Figure 3:**
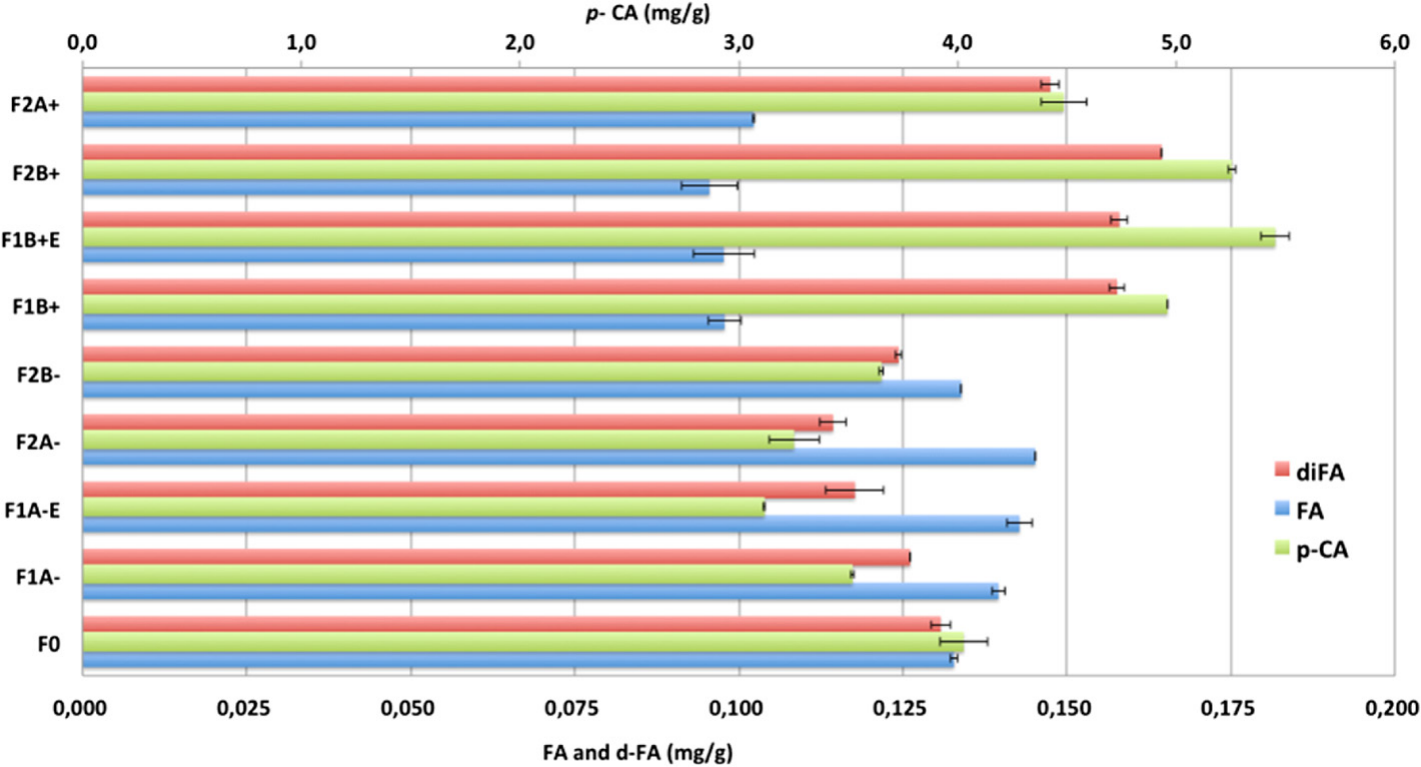
**Yield (mg g** ^**−1**^ **) of** ***p*** **-coumaric acid, ferulic acid and di-ferulic acid in different WS fractions.** diFA: di-ferulic acids (ferulic acids dimer); FA: Ferulic acid; *p*-CA, p-coumaric acid.

Ultrafine milling combined with ES technology resulted in the separation of the high crystalline cellulose F– and low crystalline cellulose F+ of WS without adding chemical catalysts and solvent extraction. The successive refineries also influenced the biochemical composition of the positively F+ and negatively F– charged fractions, as shown in Table 1 and Figure 3. It can be observed that the positively charged fractions F+ were richer in cellulose compared to F0 and negatively charged F– fractions. Whereas, negatively charged F– fractions were rich in lignin, hemicelluloses (and thus arabinoxylans) and ash compared to positively charged F + fractions. This corresponds to hem:cell ratios of 0.77, 0.82, 0.68 and 0.81 for F1A–, F2A–, F2B– and F1A–E compared to 0.45, 0.37, 0.49 and 0.37 for F1B+, F2B+, F2A+ and F1B+E, respectively and to 0.66 for F0 (Table 1).

ES is a very effective technology to isolate or separate fractions enriched in various biopolymers or byproducts without solvent utilization and effluent production. For instance, in the case of F2B+ fraction, an increase of 33% for cellulose and decrease of 30% for hemicelluloses were observed compared to the starting material F0 (Table 1). Also, it can be seen in Table 1 that F1A–E fraction (the material that was found stuck to the electrodes) exhibited a very singular composition, characterized by 30.3, 16.7 and 37.6% of hemicelluloses, lignin and cellulose, respectively. Moreover, it exhibited a very high degree of cellulose crystallinity (63.5%), high reactive surface (70.7 m^2^/g) and high ash content (15.3%) compared to the unfractionated WS F0 and to other fractions (Table 1). Figure 3 also shows that the negatively charged fractions F– are richer in ferulic acid (FA), which is known to be a characteristic of the complex lignin-xylan in the gramineous lignocellulosic plants. In contrast, the positively charged fractions F+ are richer in *p*-coumaric acid (*p*-CA) and ferulic acid dimer (diFA).

Table 1 and Figure 3 show that at each fractionation or refinery step (Figure 1c) the positively charged particles contained more cellulose and phenolic acids (*p*-CA and diFA) than the negatively charged particles and the starting material F0. This corresponded to the observed increase in cellulose content in the positively charged fractions; 55.2% after the first fractionation step and 58.4% after the second fractionation step. The successive refineries steps also resulted in an increase in the content of hemicellulose and FA in the negatively charged fractions and in a decrease of cellulose content phenolic acids (*p*-CA and diFA). The difference in color, microstructure, biochemical composition and structure could depend on the origin of the tissues, which clearly indicates that using ES, would make it possible to separate different lignocellulosic tissues and fractions, displaying very different structures and biochemical compositions.

### Enzymatic hydrolysis of wheat straw fractions and energy efficiency

**Figure 4:**
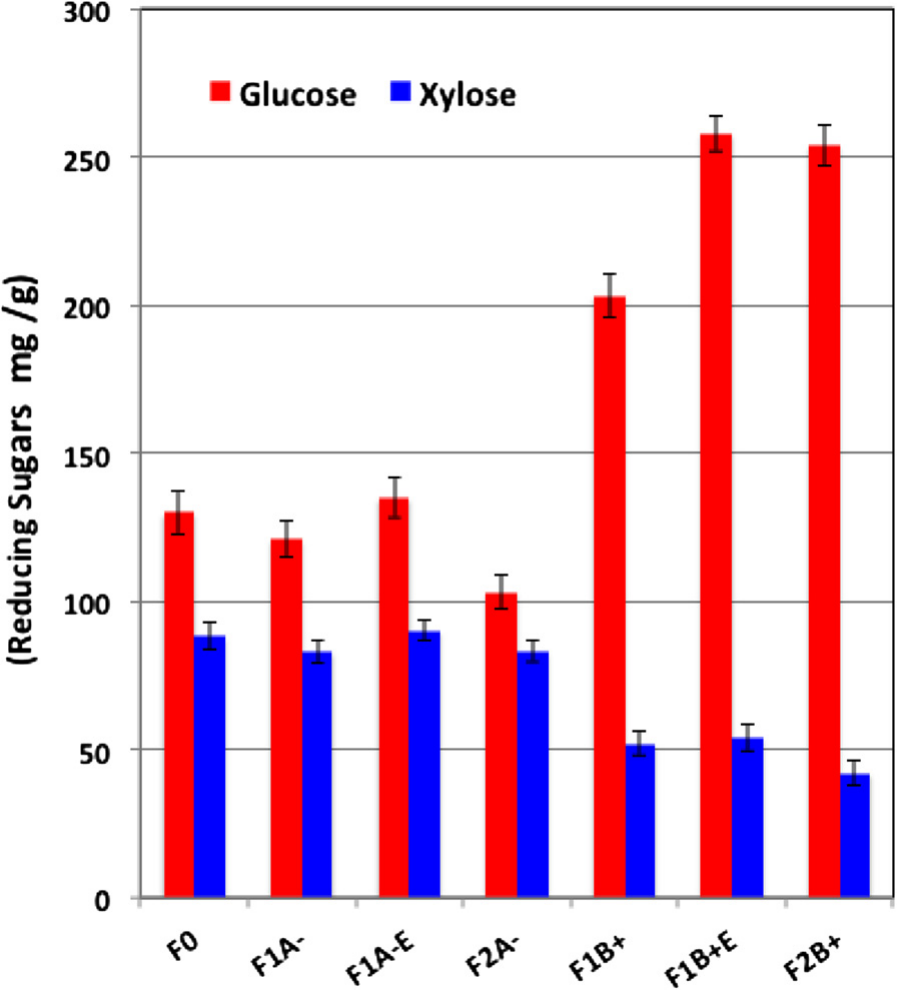
**Glucose and xylose yield (mg g** ^**−1**^ **) after enzymatic hydrolysis of different fractions.**

After each separation step, WS fractions were hydrolyzed with a commercial enzymatic cocktail at biomass loadings of 10% in buffer and enzymatic loadings of 20 FPU (filter paper unit) g^−1^ for 72 hours [10]. The effects of each separation step (Figure 1 and Figure 4) were evaluated by determining the glucose and xylose released (mg g^−1^ fraction). The data presented in Figure 4 illustrate that the maximum glucose yield after 72 hours was obtained with positively charged fractions F+, with a yield of about 258, 254 and 203 mg glucose g^−1^ of F1B+E, F2B+ and F1B+ fractions, respectively. There was a highly significant difference observed in the glucose and xylose yield obtained for positively charged fractions F+ compared to negatively charged fractions F– and starting material F0. The glucose yields after 72 hours of hydrolysis was 130, 121, 135 and 103 mg g^−1^ of F0, F1A–, F1A-E and F2A– respectively. Whereas, the maximum xylose yield was obtained with negatively charged fractions F–, with a yield of about 83, 90 and 83 mg xylose g^−1^ of F1A–, F1A-E and F2A– respectively, compared to 88 mg xylose g^−1^ of F0 and 52, 54 and 42 mg xylose g^−1^ of F1B+, F1B+E and F2B+ respectively. These results suggest that the positively charged fractions F+ are more accessible to cellulase than the negatively charged fractions F–.

ES technology has allowed for very effective isolating and separating of poor enzymatically accessible or recalcitrant tissues F– from the more accessible tissues or fractions F+. In comparison to another mechanical treatment developed in literature (Table 2), Maache-Rezzoug *et al*. [26] compared the effect of mechanical treatment on glucan conversion, and found that glucose yield increased with a decrease in particle size [26]. They obtained 120 and 150 mg g^−1^ WS of glucose with particle sizes of 600-1000 μm and 50-600 μm, respectively. Silva *et al*. [27] also investigated the effect of mechanical treatment on enzymatic hydrolysis of WS and obtained about 108 mg g^−1^ WS of reducing sugars with a mean particle size of 270 μm, which was obtained with centrifugal milling, and the maximum reducing sugars yield was 240 mg g^−1^ WS obtained with a particles size of 10 μm, produced by ball milling for 240 hours (Table 2). This indicates that the accessibility does not depend only on the particle size and/or the cellulose crystallinity, but also on parameters such as the reactive SA, lignin and ash contents, and lignin-xylan association [7,25,28,29].

**Table 2:** **Comparison of various WS pretreatments with dry fractionation technology developed in this work**

**Pretreatment conditions**	**Enzymatic hydrolysis conditions**	**Glucose (mg/g WS)**	**Reducing sugars (mg/g WS)**	**Solvent or water (L/Kg WS)**	**Chemical reagent (Kg/Kg WS)**	**Reference**
Mechanical						
50 - 600 μm	Celluclast-1.5 L *Trichoderma reesei*, 50°C, 20 hours	150	ND	0	0	[27]
600 - 1000 μm		120	ND	0	0	
Ball milling 240 hour, 10 μm	Cellulase *Trichoderma reesei*, 37°C, 48 hours	N.D.	270	0	0	[28]
Centrifugal milling: 270 μm		N.D.	108	0	0	
Milling + electrostatic fractionation: 50 -70 μm	Cellulase *Trichoderma Longibrachiatum* 37°C, 72 hours	254*	294*	0	0	This study
Chemical						
750 μm, glycerol 70%, 230°C, 4 hours	Celluclast-1.5 L *Trichoderma reesei*, 50°C, 2.5 h	268	423	20	0	[30]
750 μm, ionic liquid/WS ratio: 25/1 w/w, 150°C, 1 hour		368	744	0.03	25	
750 μm, 1% acid, 140°C, 40 minutes		176	223	20	1	
Physicochemical						
833 μm, water/WS ratio: 40/1, Steam explosion: 210°C, 10 minutes	Cellulase BTXL *Trichoderma reesei*, 50°C, 72 hours	ND	117	40	0	[31]
833 μm, 50% v/v acetic acid, steam explosion: 220°C, 8 minutes		ND	244	40	20	
833 μm, 70% v/v ethanol steam explosion: 220°C, 5 minutes		ND	264	40	-	
833 μm, supercritical CO_2_ 190°C, 12 MPa, 30 minutes	Cellulase *Trichoderma reesei*, 50°C, 72 hours	ND	149	ND	ND	[32]
833 μm, steam explosion (A) 200°C, 15 minutes + supercritical CO_2_: 190°C, 12 MPa, 30 minutes		ND	234	ND	ND	

ND not determined; WS: wheat straw.

*254 gKg^−1^ of glucose and 294 gKg^−1^ of reducing sugars obtained for F2B+ fraction after ES.

In this study, we confirm this hypothesis - that negatively charged fractions F– or tissues characterized by a heterogeneous and coarse particle content, richer in lignin-xylan and ash, and with more crystalline cellulose, are less accessible and produce lower glucose yields compared to initial F0 and to positively charged fractions F+, characterized by high reactive SA and fine particle size, a lower lignin content and less crystalline cellulose (Table 1). The combination strategy of simultaneous ultrafine milling and ES improve the economic feasibility by low energy consumption (10.5 WhKg^−1^) compared, for example, to ball milling and steam or hot water, which consumes high energy. ES technology also produced different tissues and fractions with different physicochemical structures and properties in a short time, which can be easily converted to biofuels and byproducts without using chemicals and/or water and without generating toxic effluent.

### Comparison of electrostatic separation technology efficiency with the others wheat straw pretreatments

The energy efficiency ɳ (g glucose extracted Wh^−1^) was used to compare the performance of lignocellulose pretreatments [2,18]. The amount of glucose recovered after enzymatic hydrolysis (g glucose Kg^−1^ biomass) was divided by the total energy consumed for fractionation or pretreatment processes (WhKg^−1^ biomass). However, literature concerning the energy consumption and energy efficiency of chemical, physicochemical and mechanical treatment is scarce. The total energy consumption (E_tot_) of the fractionation technology developed in this current work has been calculated from Equation 1:

$$ {\mathrm{E}}_{\mathrm{tot}}={\mathrm{E}}_{\mathrm{M}}+{\mathrm{E}}_{\mathrm{E}\mathrm{S}} $$

Where EM is the specific energy consumption required during the milling of WS using screen size of 0.1 mm, and E_ES_ is the specific energy consumption during the ES fractionation of WS (fraction F0).

The specific energy requirement (E_M_) to milling raw material using an impact knife mill (Figure 1a) with 0.1 mm screen sizes for producing F0 fraction was 125.7 WhKg^−1^. In contrast, only 10.5 WhKg^−1^ was required for positively charged F+ and negatively charged F– fractions production from F0 fraction (Figure 1b and c). This clearly indicates that ES technology consumes less energy compared to milling equipment used to reduce particle size, such as knife and ball milling, and to thermal pretreatments, such as steam and hot water [7,33,34]. As a consequence, the combination of ultrafine milling and ES for the production of positively and negatively charged fractions consumed approximately 136.2 WhKg^−1^ (E_tot_). The total energy requirement (E_tot_) was used to calculate the energy efficiency (ɳ). The highest ɳ was the more effective pretreatment or of biomass. The maximum glucose yield extracted was 258 g Kg^−1^ of F1B+E and 254 g Kg^−1^ of F2B+ and after ES and hydrolysis, whereas only 130 g Kg^−1^ of F0 was obtained of unfractionated WS. The highest ɳ obtained was 1.86 g glucose Wh^−1^ for F2B+E and F2B+ compared to 1.03 g glucose Wh^−1^ for unfractionated WS (F0), representing a gain of 0.70 g glucose Wh^−1^. In comparison, Silva *et al*. [27] reported that the maximum reducing sugars yield, obtained with BM at 240 hours, was 240 mg g^−1^ WS, consuming more energy than the combination of milling and ES technology developed in this study [7,33].

In comparison, the energy efficiency of acid and alkaline pretreatment of oilseed rape (OSR) straw was evaluated [35]. The maximum sugar extracted after acid pretreatment and hydrolysis was obtained from biomass pretreated for 90 minutes. However the ɳ was found to be higher when biomass has been pretreated for 60 minutes; hence, the highest ɳ obtained was 0.94 g glucose Wh^−1^ from a pretreatment time of 60 minutes. However, during alkaline pretreatment, the highest glucose yield (462 g glucose Kg^−1^ biomass) was obtained by pretreating biomass for 30 minutes at 0.63 mol/dm^3^ NaOH concentration [35]. The highest ɳ obtained after alkaline pretreatment was 1.42 g glucose Wh^−1^ by pretreating biomass for 30 minutes at 130°C and a NaOH concentration of 0.63 and 0.75 mol/dm^3^. The authors have shown that a higher glucose concentration can be extracted from OSR straw per watt-hour of energy consumed, when alkaline pretreatment was used in contrast to acid pretreatment.

da Silva *et al*. [36] studied the efficiency of wet disk milling (WDM) on bagasse and sugarcane straw for bioethanol production. They reported that the maximum sugar yields were obtained after 20 cycles WDM for both bagasse and straw, which yielded 213 and 245 g glucose Kg^−1^ biomass, respectively. However, the highest ɳ obtained was 0.046 and 0.027 g glucose Wh^−1^, for bagasse and straw biomass, respectively after only 10 cycles of WDM, while 20 cycles consumed the highest amount of energy, corresponding to the lowest ɳ. Hideno *et al*. [37] also compared the efficiency energy of BM, WDM and hot compressed water treatment HCWT. They suggested that the optimal milling time was 60 minutes with the highest yield of glucose (331 mg glucose g^−1^ RS). However, BM treatment at 60 minutes resulted in lower ɳ compared to WDM at 5 minutes and 10 minutes for the pretreatment of RS. The highest ɳ obtained was 0.078 g glucose Wh^−1^, for RS after BM at 5 minutes. The HCWT of RS were performed at 160°C and 180°C. The glucose yield after enzymatic hydrolysis at 180°C was 313 mg glucose g^−1^ RS, higher than that at 160°C and higher than WDM treatment for one cycle. But in terms of energy efficiency, a WDM pretreatment of one cycle is more preferable than HCWT at 180°C [37]. These results clearly indicate that energy efficiency is an important parameter that can be used in the comparison of the efficiency of different lignocellulosic pretreatments.

In Table 2, we reported a different WS pretreatment; however, it is very difficult to carry out an in-depth comparison because most published studies were conducted under a wide variety of conditions. As a consequence, the comparison of the combination of dry milling and ES technology developed in this study with ionic liquid or dilute acid and steam explosion pretreatments was not evident (Table 2). A global harmonization initiative is needed. It can be seen in the Table 2 that the electrostatic fractionation technology of WS developed in this work required low energy (10.5 Wh Kg^−1^) and produced a high glucose yield (254 mg g^−1^) without using chemicals and without wastewaters. This can be compared to 150 mg g^−1^ obtained with a mechanical treatment [26], 268 mg g^−1^ WS obtained with a particle size of 750 μm using 70% glycerol at 230°C for 4 hours [38], 368 mg g^−1^ using ionic liquid with a liquid/WS ratio of 25/1 w/w (25 Kg ionic liquid Kg^−1^ WS) at 150°C for 1 hour [38], 176 mg g^−1^ with a particle size of 750 μm using 1% of sulfuric acid at 140°C for 40 minutes [38], and 264 mg g^−1^ of total sugars obtained with a particle size of 833 μm after steam explosion using ethanol 70% v/v at 220°C for 5 minutes [30]. Some of these different studies developed on WS used a high quantity of water, chemical reagent and solvent and generated high levels of effluent and produced fermentation inhibitors, which have a severe environmental impact (Table 2). The simultaneous combination of milling and electrostatic fractionation of lignocellulose biomass could improve the economic feasibility by low energy consumption and produce reactive lignocellulose particles with different physicochemical structures in a short time, which can be converted easily to biofuels without using chemicals or water and without generating toxic effluent.

We conclude from this discussion that in order to compare or to evaluate the efficiency and the performance of different lignocellulose pretreatments and fractionation methods some parameters are necessary, such as: the energy requirement to reduce the particle size; the energy consumption during thermochemical treatments and drying process; glucose or reducing sugars yield; the energy consumed during separation, extraction, and solvent recycling; and the quantity of chemical reagent, water and solvent used in the pretreatment.

## Conclusions

The combination of milling and ES of WS as a continuous dry fractionation process produced interesting fractions which exhibit a very different structure and composition. Homogeneous and crumbly fractions rich in cellulose were clearly more abundant in the positively charged particle fractions, and heterogeneous and fibrous fractions rich in lignin-hemicelluloses and ash were more abundant in the negatively charged fractions. The positively charged fractions are more accessible to enzymes, permitting a high glucose yield, which could be used as substrate for biofuels. However, the negatively charged fractions are more recalcitrant and contained more lignin-xylan and ash, which are less accessible to enzymes. The combination of milling and ES technology appears to be an interesting new fractionation continuous process for the development of environmental lignocellulosic biorefinery for biofuels and byproducts or biomaterials production, without using chemical and without wastewaters generation.

## Materials and methods

### Fractionation of wheat straw

WS was obtained from a local farm (Languedoc-Roussillon region, France). WS was coarsely cut to less than 2 mm by knife milling SM 100 (Retsch, Germany). WS sample (2 mm particle size) was also ground using an impact knife mill (Hosokawa-alpine, type UPZ, Augsburg, Germany, Figure 1a), operated at ambient temperature at a speed of 18000 rpm, with a 0.1 mm screen size (Figure 1a and b). After milling, the unfractionated WS powder (F0) was introduced directly into a Tribo-electrostatic Separator Pilot (TEP System, Lexington, United States; Figure 1a) is shown in Figure 1a and b. The particles were conveyed by compressed air at 1 Kg h^−1^ in a charging line where they were tribo-charged, by impacting each other and impacting against the walls of the charging line (Figure 1a and b). The charged particles were then injected into a separation chamber containing two high voltage electrodes (10000 V), where the positively charged particles are attracted by the negative electrode and the negatively charged particles are attracted by the positive electrode (Figure 1b). A particle recovery system equipped with two cyclones allowed for the separate recovery of the two fractions (one containing the positively charged particles and the other, the negatively charged particles). These two separated fractions underwent a second separation step, giving four different fractions (Figure 1c).

When the starting material was F0 only two separation steps were carried out: the fractions F1A– and F1B+ were obtained from F0 as a result of the first separation step, while the fractions F2A– were obtained from F1A–, and the fractions F2B+ were obtained from F1B+ as a result of the second fractionation step (Figure 1c).

The total energy (E_tot_) consumed during the fractionation process, defined as the sum of E_M_ and E_ES_, was calculated. The total energy consumption (E_tot_ in WhKg^−1^) was measured according to Equation 1. E_M_ and E_ES_ are measured using a wattmeter PX110 (Power meter, Meteix, France). The power active, active electric energy (Wh), frequency hertz and time were logged into a PC card at 1-second intervals [10].

### Characterization of wheat straw fractions

WS sample (F0, F1A–, F2A–, F2B–, F1A–E, F1B+, F2B+, F2A+ and F1B+E) were analyzed for their particle size, color, biochemical composition, crystallinity and microstructure and enzymatic accessibility. The particle size was analyzed by laser granulometry Mastersizer 2000 (Malvern Instrument, Orasy, France) and the particle density was determined using ultra Pycnometer 1000 (Quantachrome Instrument, United States). The carbohydrate and lignin composition of lignocellulosic samples was measured after concentrated acid hydrolysis according to the method described by Barakat *et al*. [10]. Phenolic acids were also analyzed using the method described by Antoine *et al*. [31]. Ester-linked phenolic acids were saponified under argon (oxygen-free) at 35°C in 2N sodium hydroxide (Sigma Chemical Co., St Louis, United States) An internal standard (2,3,5 trimethoxy-(E)-cinnamic acid, T-4002, Sigma Chemical Co., St Louis, United States) was added before adjusting the pH to 2. Phenolic acids 371 were then extracted with diethylether and quantified by high-performance liquid chromatography. The response factors of *p*-CA, vanillic acid (VA), FA and diFA were determined at 320 nm with purified samples, relative to the internal standard. The FA monomer content was calculated from the amount of cis- and trans-ferulic acid.

The crystallinity of different WS fractions was determined by X-ray diffraction. Powder X377 ray diffraction patterns were recorded on a Bruker diffractometer D8 Advance (Bruker corporation, Germany)**.** The measurements were conducted on powder-compacted on small mats. X-Ray-diffraction data were collected from 2θ = 5 to 50° with a step interval of 0.02°. The degree of crystallinity can be expressed as the percentage crystallinity index [10].

### Enzymatic hydrolysis

Enzymatic hydrolysis of treated and untreated WS was performed using a commercial enzymatic cocktail (*Trichoderma longibrachiatum* C9748) (20 FPU g^−1^) obtained from Sigma Aldrich (Sigma Chemical Co., St Louis, United States). Enzymatic hydrolysis was conducted at a solid concentration of 10% (w/v) in 50 mM sodium acetate buffer (pH 5.0) at 37°C for 72 hours with agitation. Sodium azide (Sigma Chemical Co., St Louis, United States) was added at the end of the experiment to inhibit microbial growth. The experiment was performed in triplicate. The enzymatic digestibility was evaluated from the obtained soluble sugars (mg g^−1^) determined by high phase liquid chromatography analysis [10].

## Abbreviations

ɳ: Energy efficiency
BM: Ball milling
CrI: Crystallinity index
E: Energy
ES: Electrostatic separation
E_M_: Energy consumption required during milling
E_ES_: energy consumption during ES
FA: Ferulic acid
p-CA: p-coumaric acid
SA: Surface area
WDM: Wet disc milling
WS: Wheat straw
VA: Vanillic acid
